# Impact Factor Evolution of Clinical Neuroradiology

**DOI:** 10.1007/s00062-023-01353-4

**Published:** 2023-11-16

**Authors:** Martin Bendszus

**Affiliations:** Neurologische Klinik, Abteilung für Neuroradiologie, Universität Heidelberg, Heidelberg, Germany

**Fig. 1:**
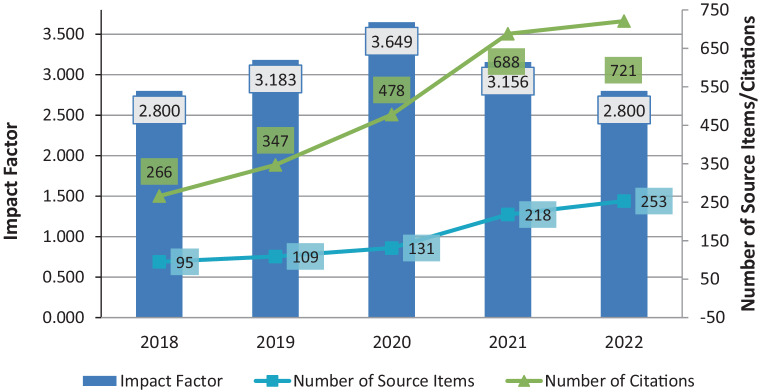
Development of the journal impact factor of *Clinical Neuroradiology* in recent years

In June 2023, the new impact factor was published. Overall, many medical journals dropped in their impact factor due to a different calculation basis in the last 2 years which will be unchanged from now on, though. *Clinical Neuroradiology* also slightly dropped with the impact factor from 3.156 to 2.8 (Fig. 1).

Most importantly, the journal remained stable in the comparative ranking of journals in radiology, nuclear medicine and medical imaging or clinical neurology (Table 1).

**Table 1:** Development of subject index rankings of *Clinical Neuroradiology* in recent years

Category name	2021	2022
Clinical neurology	120/212	121/212
Radiology, nuclear medicine and medical imaging	72/136 (Q3)	67/135

**Fig. 2:**
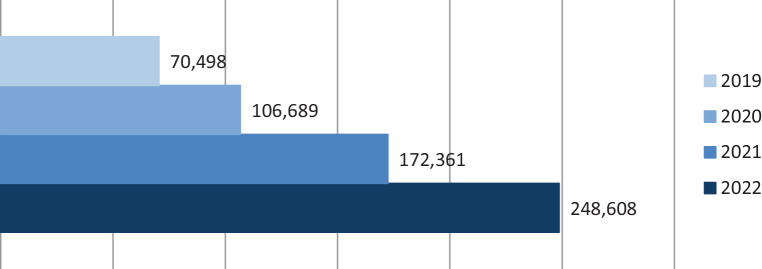
Development of article downloads at *Clinical Neuroradiology* in recent years

Therefore, the slight drop in impact factor is caused by the overall changed calculation basis. Looking at the number and quality of submitted manuscripts, we are confident that in 2024 the journal’s impact factor will rise again. The journal’s growing impact is also underscored by the increasing number of downloaded papers of *Clinical Neuroradiology *(Fig. 2).

Finally, it is noteworthy that the relevance of a journal is not only reflected by the impact factor (which is a simple calculation of published and cited manuscripts) but many other factors.

We would like to thank all authors and reviewers for their continuous support of *Clinical Neuroradiology *to establish this journal as one of the leading journals in the field.

Martin Bendszus

Editor-in-Chief

